# A New Dental Depot

**Published:** 1862-08

**Authors:** 


### A NEW DENTAL DEPOT.

By reference to our advertising sheet, it will be observed that a new Dental Depot has been established at No. 11 North Ninth Street, Philadelphia, by Rubencame & Stockton. These gentlemen are furnishing the profession with choice goods in the dental line. They manufacture teeth of a very superior quality, and dental instruments and appliances of all kinds. From the stand they have taken, they must succeed; and we most heartily recommend them to the profersion, knowing that everything will be done by them to render perfect satisfaction. T.

